# Peri-personal space as a prior in coupling visual and proprioceptive signals

**DOI:** 10.1038/s41598-018-33961-3

**Published:** 2018-10-25

**Authors:** Jean-Paul Noel, Majed Samad, Andrew Doxon, Justin Clark, Sean Keller, Massimiliano Di Luca

**Affiliations:** 1 Oculus Research, Facebook Inc., Redmond, WA USA; 2 Vanderbilt Brain Institute, Vanderbilt University, Nashville, TN USA; 3 Department of Psychology, University of California Los Angeles, Los Angeles, CA USA; 4 Centre for Computational Neuroscience and Cognitive Robotics, University of Birmingham, Birmingham, UK

## Abstract

It has been suggested that the integration of multiple body-related sources of information within the peri-personal space (PPS) scaffolds body ownership. However, a normative computational framework detailing the functional role of PPS is still missing. Here we cast PPS as a visuo-proprioceptive Bayesian inference problem whereby objects we see in our environment are more likely to engender sensations as they come near to the body. We propose that PPS is the reflection of such an increased *a priori* probability of visuo-proprioceptive coupling that surrounds the body. To test this prediction, we immersed participants in a highly realistic virtual reality (VR) simulation of their right arm and surrounding environment. We asked participants to perform target-directed reaches toward visual, proprioceptive, and visuo-proprioceptive targets while visually displaying their reaching arm (body visible condition) or not (body invisible condition). Reach end-points are analyzed in light of the coupling prior framework, where the extension of PPS is taken to be represented by the spatial dispersion of the coupling prior between visual and proprioceptive estimates of arm location. Results demonstrate that if the body is not visible, the spatial dispersion of the visuo-proprioceptive coupling relaxes, whereas the strength of coupling remains stable. By demonstrating a distance-dependent alteration in visual and proprioceptive localization attractive pull toward one another (stronger pull at small spatial discrepancies) when the body is rendered invisible – an effect that is well accounted for by the visuo-proprioceptive coupling prior – the results suggest that the visible body grounds visuo-proprioceptive coupling preferentially in the near vs. far space.

## Introduction

A large proportion of human interactions with the environment are mediated by the body and as such occur within the peripersonal space (PPS), the volume of space that surrounds and is immediately adjacent to the body^1^. Because of the evolutionary importance of the space surrounding the body, it is no surprise that a dedicated fronto-parietal multisensory network has evolved to process information preferentially for the space near the body. Early neurophysiological work in non-human primates^2^ demonstrated that this space possesses peculiar multisensory properties as it is encoded by visuo-tactile^3^ and audio-tactile^4^ neurons that have body-centered or body-part-centered reference frames^5,6^. Furthermore, these neurons preferentially respond to approaching stimuli^7,8^ and they trigger predefined defensive responses when electrically stimulated^9,10^. Taken together, these findings have led to the idea that the function of PPS is twofold: it serves both as a protective buffer zone between an organism and its environment^11^ and as the interface between perception and action in the manipulation of the environment by a part of the body^12,13^.

There is converging evidence supporting the existence of a PPS system in humans. Neuropsychological investigations of patients showing distance-dependent cross-modal extinction^14,15^, psychophysical results demonstrating facilitated tactile processing when exteroceptive signals are presented near the body^16–21^, and neuroimaging findings^22^ are all consistent with the presence of a PPS representation in humans. There is a good correspondence between the networks underpinning PPS both in monkeys and humans^23,24^ and the psychophysical studies have both replicated and extended the early neurophysiological understandings. Namely, human PPS has been demonstrated to exist both over specific body parts and as a global feature of the body as a whole^25^. Further, the PPS representation in humans, as in non-human primates, appears to be particularly tuned to approaching dynamic stimuli^26,27^. Lastly, and again comparably to what happens with non-human primates^28^, human PPS adaptively recalibrates during tools use^29^ and remaps both following performed actions^18^ and as a consequence of action possibility^13,17,21,30,31^.

Interestingly, recent influential theoretical accounts of body ownership^32–34^ and empirical findings^20,35^ suggest that PPS may be a stepping-stone toward embodiment (see^36^ for a similar finding in non-human primates). In support of this view, PPS has been documented to extend over the perceived location of body parts and not their veritable physical location^20,35^, and the neural substrates of PPS and body ownership have been shown to be largely overlapping^37^. Likewise, the rubber-hand illusion (RHI)^38^, a multisensory illusion during which one feels ownership over a fake hand, can be elicited solely within the boundaries of one’s PPS^39,40^.

Arguably, however, more direct examinations of the relationship between the visually represented body and the multisensory PPS are largely lacking due to i) the fact that PPS has traditionally been studied in the real world and via reaction time paradigms that necessitate the presence of the body, and ii) the difficulty – or impossibility, without novel virtual reality (VR) technology - to study PPS while not displaying a body yet concurrently leaving the rest of the visual scene unchanged. In rare exceptions and making use of VR setups, D’Angelo and colleagues^41^ have recently observed that body invisibility induces a contraction of interpersonal space, yet not of the judgement of their reaching limit, while Guterstam and colleagues have suggested that whole-body^42^ or body-part^43^ ownership over an empty space is possible. Given these results, in a first aim here we sought to further probe the putative relation between bodily self-consciousness and PPS by examining whether the latter representation was “preserved yet modified” when the body was rendered invisible. This specific hypothesis is supported by the above-mentioned findings that embodiment, putatively reliant on PPS encoding^20,33–35^, over empty spaces is possible^42,43^, yet interpersonal space – a concept closely related but not identical to PPS (see see Clery & Ben Hamed^44^ and Hunley & Lourenco^45^) – is contracted^41^ when the body is rendered invisible.

Further, and perhaps most vexingly, while there are neural network models accounting for phenomena related to PPS^8,46,47^ there is no computational framework accounting for its functional role. In recent years Clery and colleagues^44,48–51^ have demonstrated that the PPS network is heavily involved in impact prediction, and a number of researchers have suggested that the RHI^38^ can be elicited even without touch^52,53^ – thus implying a touch prediction role to the PPS. More directly, Guterstam and colleagues have reported that when brushstrokes are applied in mid-air near participants hands concurrently with touch on their real hand, subjects report feeling a “magnetic touch illusion”^54^. Further, in line with PPS encoding, this illusion shows a non-linear decay at approximately 40 cm from the hand^54^, and this area of space increases following tool-use^55^. In turn, Guterstam and colleagues suggest that the “magnetic touch illusion” is a perceptual correlate of visuo-tactile integration in the PPS^54^, and taken together, these results imply that the PPS allows for the inference of putative touch by nearby objects. In turn, aiming to develop a computational framework accounting for the functional role of PPS, here we argue that PPS can be conceived as a “stochastic spatial bubble” surrounding the body wherein the probability that objects in the world could come into contact with the body is computed (see^44,48,49,51,56,57^ for similar arguments). Arguably, the PPS may allow for the computation of the probability that sensory signals are associated with each other and most importantly with one’s body. Such probability may be computed in 3D space and, since the body can move, these probabilistic computations ought to follow the body movements in real time (or even predict them, see^13,30,31^). In this work, we put forward a novel theoretical framework for PPS that estimates the increasing tendency to couple body-related exteroceptive signals (i.e. visual) as these approach the body (i.e., the source of tactile and proprioceptive signals).

In a way similar to Samad and colleagues^53^, who expressed the RHI as the result of a Bayesian Causal Inference process^58^, here the concept of PPS is casted within a Bayesian framework where visual and proprioceptive sensory signals about one’s hand (i.e., the likelihoods) are combined with the *a priori* probability of all possible locations for those sensations. This combination of sensory likelihoods and the prior probability of signal sources estimates the posterior probability of the hand position. In this scheme, the PPS is expressed as the spatial coupling prior capturing increased probability that visual and proprioceptive signals are located close to each other. Such probability changes the way multisensory signals are integrated/segregated and, because of this influence, the impact of PPS on perception may be indexed by using a task that measures the attraction of signals toward the expected mapping with different incongruences between multisensory signals (much as in Guterstam *et al*.^54,55^ with the “magnetic touch illusion”). We can estimate the shape of the *a priori* visuo-proprioceptive coupling by asking participants to indicate where they perceive their finger (e.g., their left index) as well as visual cues in the environment. Importantly, by using state of the art virtual reality technology, we can also either visually render the participant’s hand that was used to give an answer (i.e. the right hand with all its 26 degrees of freedom, sub-millimeter spatial resolution and imperceptible temporal lag; see http://goo.gl/YRaEPX) or we can visually render the rest of the environment without showing the hand that was used to answer. In turn, this manipulation permits a direct examination of the relationship between the visibility of the body and the perceptual effects due to PPS. In other words, by manipulating the visibility of the answering hand we can address the following question: does an invisible body still have a dedicated multisensory spatial representation surrounding it? Furthermore, by employing the above-mentioned experimental design and attempting to account for the observed localization data via a Bayesian coupling prior framework, we can test our conceptual model suggesting that the PPS represents a stochastic bubble surrounding the body and computing the likelihood that objects will come in contact with the body.

## Methods

### Participants

Twenty right-handed subjects (11 females, mean age = 33.1 years old, range = 22–50 years old) took part in the experiment. Explicit power calculations were not performed prior to initiating data collection due to the paucity of reports studying body representation during invisibility (and complete lack of studies pertaining to visuo-proprioceptive coupling during invisibility). However, a stopping rule of N = 20 was established prior to data collection, as this sample size is well in line with the majority of studies regarding visuo-proprioceptive integration and PPS representation (e.g., see^17–21,25,59–61^). Participants had normal or corrected-to-normal visual acuity, self-reported normal hearing and somatosensation, and no history of neurological or psychiatric disorder. All participants gave informed written consent before taking part in the experiment, which was conducted according to the protocol approved by Western Institutional Review Board (WIRB). Participants were remunerated for their time.

### Materials and Apparatus

**Figure 1:**
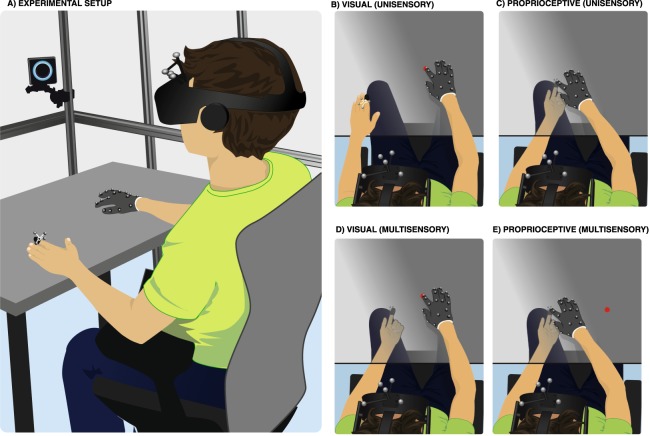
Experimental Setup and Paradigm. (**A**) Experimental Setup; participants wore a Head-Mounted Display, as well as a mesh glove on their right hand (reaching hand) and a thimble on their left index finger (target finger), all whom were tracked via 17 OptiTrack cameras (1 depicted). This setup allowed for hand tracking both above and below the table. Participants performed four types of reaches (**A**–**D**) either in unisensory or multisensory blocks of trials. (**B**) On visual target trials in unisensory blocks, a red dot appeared on the table in the virtual environment, and participants were to reach toward this target with their right (reaching) hand. The left hand was placed at its home position. (**C**) On proprioceptive target trials in unisensory blocks, participants were to place their left index finger at a pre-determined location under the table, as guided by arrows pointing in the direction of the pre-determined location (not depicted). Once the arrows disappeared, participants reached toward the proprioceptive target. (**D**,**E**) in multisensory blocks, participants placed their left finger under the table at a pre-determined location and a red dot appeared either congruently (directly above) with the proprioceptive target, or at a spatial disparity in the frontoparallel plane (i.e., no depth disparity). Participants were first to reach toward the visual target (**D**) and subsequently toward the proprioceptive target (**E**).

Participants sat at a table (100 cm horizontal × 60 cm depth) laminated with felt cloth positioned inside a tracking cage (186 cm horizontal × 212 cm vertical × 157 depth) on which 17 OptiTrack cameras (Prime 17 W, 1664 × 1088 pixels, 70° FOV, 120 FPS, 2.8 ms latency) were placed to track the participant’s hands both below and above the table (see Fig. 1A). Motion capture was undertaken at 90 Hz via Motive 1.9.0 software and restricted to a volume of 110 cm horizontally × 150 cm vertically × 110 cm in depth. The participant wore a glove on their right hand (i.e., the hand used for answering) with 19 fiducial markers glued on the top surface. The hands’ natural resting positions on the proximal edge of the tabletop and in line with the shoulder were designated as their home positions. The 3D position of each of the 19 markers was reconstructed by the OptiTrack system and then sent to a near real-time hand tracking service which labeled the markers and computed a hand pose that best fit the observed markers position via a neural network. The visual rendering of the hand was created by offline capturing a single low-resolution mesh template of a male right hand via a 3D scanner (3Dmd, 3Dmd LLC, Atlanta, Georgia, USA), and then having a graphic artist overlay texture mimicking the glove utilized during the experiment in order to maximize realism. In addition to the glove on their right hand, participants wore a custom-made 3D-printed plastic thimble on their left index finger (i.e., the target finger whose position was investigated) with 4 markers and a head mounted display (Oculus Rift, 2160 × 1200 pixels, 110° FOV, 90 Hz refresh rate) with 5 markers so that they can be monitored via OptiTrack as rigid bodies defined by a unique constellation of fiducial markers. The precision of hand tracking and rendering was approximately 0.2 mm in all spatial dimensions and had a delay of 38 ms (measured according to the method described in^62^).

The virtual scene displayed on the Oculus Rift was composed of a black ground floor extending to infinity, a spatially aligned grey texture-mapped replica of the table at which participant sat (<1 mm alignment error), a visual target, and the right hand (or not, depending on experimental condition) in an otherwise black environment. Targets were randomly placed within an area 30 cm horizontally × 10 cm in depth at a minimum distance of 25 cm directly in front of the participant. The visual target was a red dot with a 1 cm diameter displayed on the surface of the table, and the proprioceptive target was the participant’s left index finger placed pointing upward on the bottom surface of the table (see Fig. 1A). To guide the participant to place the finger in a randomized position without presenting a visual counterpart of the finger position, a field of 12 blue arrows arranged on a 3 × 4 grid was rendered on the table. The size and orientation of the arrows changed continuously as a function of the relative position between the pre-determined location of the proprioceptive target and the current location of the left-index finger. Participants were instructed to shrink the arrows by moving their left finger and to keep the finger at the location where the arrow completely disappeared (within 5 mm of the pre-determined proprioceptive target position). The exact position (<1 mm error) of the right index fingertip at the end of a reaching motion was automatically recorded. The position was considered to be “final” when the following conditions were met: (1) the reaching hand had moved from its home position at least 2 cm, (2) the velocity of the participant’s reaching hand had exceeded 10 cm/s, (3) the response finger was moving slower than 0.05 cm/s for 0.5 s, and finally (4) the finger was closer than 3 cm from the table vertically (i.e., the finger was touching the surface of the table).

### Procedure

Participants initiated each trial by positioning both of their hands in their respective home positions, flat on the table with the index finger approximately 10 cm rightward (for the reaching hand) and 10 cm leftward (for the target hand) from the body midline (Fig. 1A). Then, they completed unspeeded reaches with their right (gloved) hand toward either the visual target (red dot in Fig. 1B,D) or the proprioceptive target (left index finger under the table in Fig. 1C,E), upon either presentation of the visual target or appropriate placement of the left proprioceptive target hand. A brief auditory cue indicated to participants that their response had been registered, and after completion of a trial, participants returned both hands to their home positions, so that the next trial could start 0.5 seconds after they stopped moving.

The target stimulus modality (visual, proprioceptive, or visuo-proprioceptive) was presented in a blocked fashion. In unisensory blocks, participants performed a reach toward a target and then returned to the home position. In visuo-proprioceptive blocks, participants first indicated the position of the visual target, then returned their reaching hand to the home position, and then they indicated the position of their felt left proprioceptive hand target, before returning the reaching hand to its home position (see^58,63^ for studies employing a similar protocol where reaches toward different components of a multisensory presentation are done successively and in the same order). Importantly, the relative position between the visual and the proprioceptive target was parametrically manipulated – setting the proprioceptive stimulus either 0 mm, 25 mm, 50 mm, 100 mm, or 200 mm to the left or right of the visual target (arbitrarily, negative disparity indicates proprioceptive stimuli to the left of the visual stimuli while positive values indicate proprioceptive stimuli presented to the right of visual stimuli). These visuo-proprioceptive disparities were chosen following pilot testing (e.g., demonstrated a range of visuo-proprioceptive pull) and were exclusively in the frontoparallel plane (i.e., no depth disparity). These spatial disparities are a critical manipulation, as by measuring pointing errors to visual and proprioceptive targets that are spatially discrepant we can estimate the attractive pull (in the horizontal plane) between the proprioceptive representation of the left index finger and visual stimuli as a function of distance. By including a range of disparities, the magnitude of the attractive pull can be quantitatively related to the amount of visuo-proprioceptive disparity (measured in cm), even in cases where the large disparity effectively nullifies such a pull. PPS size is known to vary with body-part size (e.g., see^25,45,63^) and be three-dimensional (i.e., spatial manipulation can be lateral, and not solely in depth). Thus, we conjectured that while at 0 mm or 25 mm discrepancy the visual stimuli was definitely within the PPS of the index finger and would lead to a measurable pull (in the case of 25 mm disparity; no disparity in 0 mm condition), at 200 mm the visual stimuli was most certainly outside the peri-finger space laterally (even if putatively within the peri-trunk space in depth). This conjecture is backed by results demonstrating no pull at 200 mm visuo-proprioceptive disparity (see Results). Further, It is important to note that although we utilized discreet values of visuo-proprioceptive discrepancies, the positions of the visual and proprioceptive targets were randomized in absolute coordinates within the space tracked. Lastly, and most importantly, participants were either shown or not shown a visual rendering of their right reaching hand.

Participants completed three types of blocks with different target stimuli modality (e.g., unisensory visual, unisensory proprioceptive, visuo-proprioceptive) for a total of 48 visual, 48 proprioceptive, and 216 visuo-proprioceptive trials. Unisensory blocks consisted of 24 trials (all either visual or proprioceptive) and multisensory blocks consisted of 18 trials (where each visuo-proprioceptive disparity was presented twice in a randomized fashion). During each block, the visibility of the reaching hand was switched from rendered to non-rendered or vice versa every 9 trials (i.e., 1 repetition per spatial disparity). Hand visibility was not randomized on a trial-per-trial basis (but in mini-blocks of 9 trials) in order to allow for potential built-up of hand ownership over the reaching hand – increasing the ecological validity of the VR scenario - which is known not to be immediate^64^. The order of target modality and visibility conditions was counterbalanced across participants. Participants were given 4 trials of practice for each target sensory modality and reaching hand visibility condition before initiation of the experiment. Overall, the experimental lasted about 60 minutes.

### Coupling Prior Model Description

**Figure 2:**
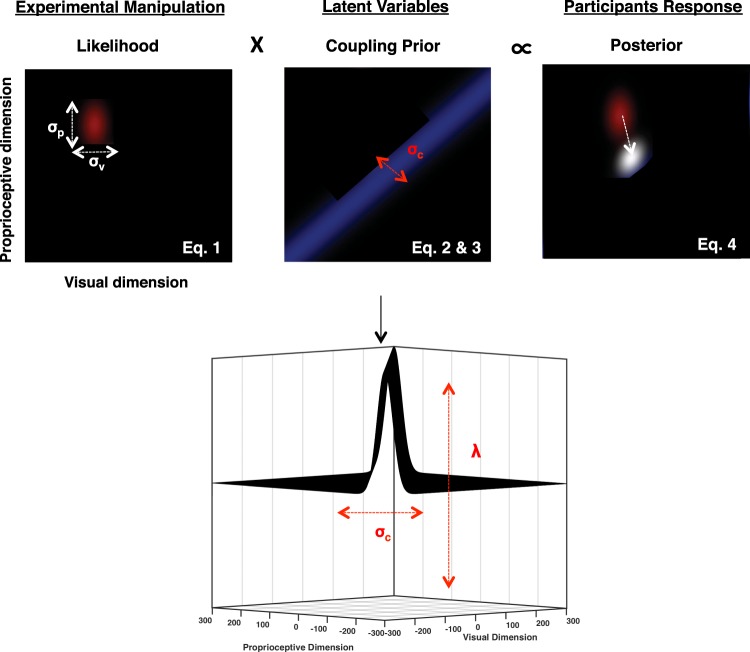
Coupling Prior Analyses. The experimental manipulation places visuo-proprioceptive targets within a two-dimensional space, which are represented by a bivariate Gaussian likelihoods with σ_p_ and σ_p_ that are calculated from individual reaches to proprioceptive and visual targets respectively (top leftmost panel). Within the coupling prior framework, this likelihood is multiplied with a prior for visuo-proprioceptive joint localization (middle panel) in order to yield the final posterior, from which a decision is decoded via maximum-a-posteriori (MAP; top rightmost panel). As illustrated in the bottom panel, the coupling prior is governed by two parameters, the spatial dispersion of the two-dimensional Gaussian describing the expected spatial relation between visual and proprioceptive targets (σ^2^_c_) and the strength of this coupling (λ), which dictates the relation between integration (larger λ), and segregation (smaller λ).

The coupling prior framework was adopted to model the reaching responses in multisensory blocks^65–69^. Each trial was regarded as the result of a computation in line with Bayes decision theory, where the likelihood probability distribution is multiplied with the coupling prior distribution to produce the posterior distribution. We determined the shape of the prior and of the likelihood so that the maximum of the posterior was located at the pointing location for each trial (Fig. 2). The likelihood function *l(v*, *p)* was modeled as a bivariate Gaussian in the space of all possible visual and proprioceptive spatial locations *v* and *p*. This likelihood had center at {µ_v_, µ_p_} imposed by the experimental manipulation (e.g., location of the visual and proprioceptive targets {m_v_, m_p_}) and its dispersion {σ_v_, σ_p_} was set equal to the standard deviation of the participant’s reaches on unisensory reaching trials (see Eq. 1):

1 $$l(v,\,p)=\frac{1}{\sqrt{2\pi {\sigma }_{v}^{2}{\sigma }_{p}^{2}}}{e}^{\frac{-{(v-{\mu }_{v})}^{2}}{2{\sigma }_{v}^{2}}+\frac{-{(p-{\mu }_{{p}})}^{2}}{2{\sigma }_{p}^{2}}}$$

The prior was modeled as the mixture of a uniform distribution whose total area can be denoted with the constant value A and the function δ(v, p), such that the prior P(v, p) was governed by the following equation:

2 $$P(v,\,p)=\lambda \,\delta (v,\,p)+(1-\lambda )\frac{1}{A}\,$$

The mixing of the two components (uniform and spatially constrained prior) arbitrates between integration and segregation of signals and is governed by the mixing parameter, λ. This latter parameter is akin to the prior for common cause within the Bayesian Causal Inference framework; reflecting the inferred causal structure of sources in the environment emitting sensory signals. The function δ(v, p) is expressed by:

3 $${\rm{\delta }}({\rm{v}},\,{\rm{p}})=\frac{1}{\sqrt{2{{\rm{\pi }}{\rm{\sigma }}}_{{\rm{c}}}^{2}}}{{\rm{e}}}^{\frac{-{({\rm{v}}-{\rm{p}})}^{2}}{2{{\rm{\sigma }}}_{{\rm{c}}}^{2}}}$$

and had maximal value (modulated by λ) at the identity line with a dispersion proportional to the parameter σ_c_. In other words, σ_c_ dictates the degree to which visual and proprioceptive cues are thought to be coupled as a function of distance, and thus in the current work we propose that this parameter represents the spatial extent of the PPS. The posterior distribution r(v, p) is obtained from the multiplication of the likelihood *l(v*, *p)*) and the prior P(v, p) at every location v and p.

4 $$r(v,\,p)=P(v,\,p)\,\ast \,l(v,\,p)$$

According to this formulation of the coupling prior model, once the values σ_v_, and σ_p_ have been determined experimentally on unisensory trials, it is possible to obtain a final estimate of the location of a bimodal stimulus {m_v_, m_p_} by finding the spatial location corresponding with the maximum of the posterior distribution, r(v, p). In the model, there are 2 free parameters, the weight of the two components of the prior λ (i.e., segregation vs. integration) and the dispersion of the coupling prior σ_c_. The free parameters were fitted by iteratively minimizing the squared difference between the participant average response and the maximum of the posterior distribution (i.e., sum of squares cost function). The λ parameter was initialized bounded between 0.01 and 0.99, and σ_c_ was bounded for each participant between the smaller of the two unisensory standard deviations and twice the maximum one. The fitting procedure was undertaken in two steps; first with a lax inter-step sum of squares decrease tolerance (*tol* = 100) and then with a much more stringent one (*tol* = 0.0001). In the first step we repeated the fitting procedure 500 times each initialized with random seeds and proceeding via gradient descent. In the second step, the 50 best-fitting parameters from the first step were then reused and the optimization was allowed to proceed until a novel and more stringent differential in sum of squares threshold was found. The parameters λ and σ_c_ from the resulting best fit were extracted for condition comparisons.

## Results

### Unisensory reaches

Reaching accuracy was analyzed by first calculating reaching error for each condition and participant separately. Subsequently, group-level analyses were undertaken in order to determine whether participants exhibited a significant bias in their reaches. Results suggested that for both visual and proprioceptive reaches in unisensory blocks there was no systematic bias (e.g., over-reaching or under-reaching) neither in the depth nor azimuthal dimension (one sample t-tests against 0, all p > 0.29). Whereas an array of research indicates that distance is largely underestimated in virtual environments^70–74^, consistent with the current findings depth estimates are considerably better in the near space^75^ and no systematic bias is observed in azimuth^76^.

A 2 (Hand visibility: Hand vs. No Hand) X 2 (Target Modality: Visual vs. Proprioceptive) repeated-measures ANOVA was performed on the magnitude (absolute value) of reaching error along the horizontal spatial dimension. This analysis demonstrated a significant main effect of hand visibility (F(1, 19) = 12.04, p = 0.003, η_p_^2^ = 0.40), wherein reaches were more accurate when the reaching hand was rendered (11.35 mm ± 2.1 mm; Mean ± Standard Error of the Mean) than when it was not (14.4 mm ± 2.3 mm). Similarly, the repeated-measures ANOVA demonstrated a significant main effect of target modality (F(1, 19) = 6.69, p = 0.019, η_p_^2^ = 0.27), wherein reaching to visual targets were generally more accurate (9.15 mm ± 2.2 mm) than reaching toward proprioceptive targets (16.7 mm ± 7.9 mm). Lastly, results showed a significant interaction between these factors (F(1, 19) = 18.01, p < 0.001, η_p_^2^ = 0.50), which was driven by the fact that proprioceptive reaches were unaltered by hand visibility (t(19) = 0.15, p = 0.87, paired-samples t-test), while reaches toward visual targets was significantly (t(19) = 6.75, p < 0.001, paired-samples t-test) more accurate when the reaching hand was rendered (5.8 mm ± 1.1 mm) than when it was not (12.5 mm ± 4.3 mm).

Similar to the case of magnitude in end-point error (i.e., accuracy), reaching standard deviation (i.e., precision) was analyzed via a 2 (Hand visibility: Hand vs. No Hand) × 2 (Target Modality: Visual vs. Proprioceptive) repeated-measures ANOVA. Reaches toward visual targets were generally more precise than those toward proprioceptive targets (9.7 ± 0.7 mm vs. 21.4 ± 2.2 mm; main effect of target modality, F(1, 19) = 30.91, p < 0.001, η_p_^2^ = 0.63). Contrarily to the case of accuracy, however, hand visibility did not generally influence precision (main effect of hand visibility: F(1, 19) = 0.52, p = 0.47). The visibility of the reaching hand, however, did differently affect the precision of reaches toward the two targets (interaction: F(1, 19) = 6.51, p = 0.02, η_p_^2^ = 0.26), significantly reducing variance toward visual targets (12.5 ± 1.1 mm vs. 6.8 ± 1.1 mm; t(19) = 3.0, p = 0.007) while not doing so for proprioceptive targets (19.8 ± 3.1 mm vs. 22.9 ± 2.0 mm; t(19) = 1.1, p = 0.28).

### Multisensory reaches

**Figure 3:**
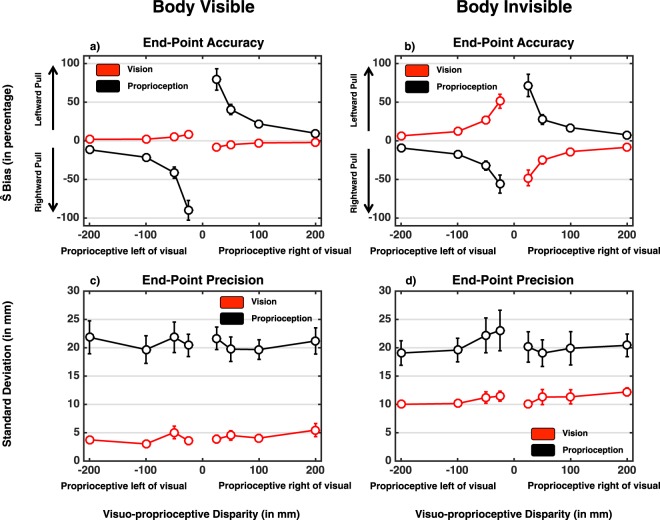
Experimental Results. (**a**,**b**) Bias in visual (red) and proprioceptive (black) location estimate (y-axis) as a function of visuo-proprioceptive spatial disparity (x-axis) and whether the reaching hand was rendered (A) or not (**b**). (**c**,**d**) Within-subject variability of the reaches toward visual (red) and proprioceptive (black) targets as a function of visuo-proprioceptive disparity (x-axis) and whether the reaching hand was visually presented (**c**) or not (**d**). Error bars represent +/−1 S.E.M.

In order to scrutinize whether the relative location (near to far; 0 mm to 200 mm) of a stimulus (visual or proprioceptive) influenced where participants pointed, we ran an initial 2 (Hand visibility: Hand vs. No Hand) × 2 (Target Modality: Visual vs. Proprioceptive) × 4 (Visuo-proprioceptive disparity magnitude: 200 mm, 100 mm, 50 mm, and 25 cm) × 2 (Disparity direction: Leftward disparity vs. Rightward disparity) repeated-measures ANOVA on endpoint reaches. In a first step (and as illustrated in Fig. 3) amalgamation of end-point reaches both at a single subject level, and then across subjects, was done on the raw (e.g., signed) error percentage in order to confirm that visual reaches were “pulled” toward the proprioceptive target, and proprioceptive end-points were “pulled” toward the visual target - see Fig. 3a,b, for confirmation that this was the case. Subsequently, in a second step and for statistical contrasts, we took the absolute value of individual subject averages (within subject averaging was done on the signed values), as here we are interested in determining whether the distance between visual and proprioceptive targets influenced the degree to which these were coupled. Using signed values would have resulted in spurious main effects and interactions of “direction” and “target modality” simply due to the sign – or direction of pull. This analysis demonstrated no main effect of direction disparity (F(1, 19) = 0.02, p = 0.87, 1 − β = 0.05), but did reveal significant main effects for disparity magnitude (F(3, 57) = 56.75, p < 0.001, η_p_^2^ = 0.75), target modality (F(1, 19) = 18.05, p < 0.001, η_p_^2^ = 0.49), and hand visibility (F(1, 19) = 11.77, p = 0.003, η_p_^2^ = 0.38; Fig. 3a,b). Overall in multisensory reaches, the bias for proprioceptive targets toward the visual target was greater (34.6 ± 5.0%) than the bias for visual targets toward the proprioceptive target (14.2 ± 1.9%) – this is in line with prediction based on unisensory and multisensory reaching precision (see below) where proprioception is less reliable in space than vision. The bias was larger when the reaching hand was not visible (26.9 ± 3.1%) than when it was visible (21.9 ± 2.9%). In fact, and most importantly as equally predicted by the fact that the standard deviation in localization estimates was selectively decreased in reaching visual targets when the hand was rendered (see Fig. 3c,d), results demonstrated a significant hand visibility × target modality interaction (F(1, 19) = 13.21, p = 0.002, η_p_^2^ = 0.41). When the reaching hand was rendered, overall bias in proprioceptive localization toward visual targets increased significantly (hand invisible, 29.8 ± 5.3%; hand visible, 39.5 ± 5.9%, paired t-test (19) = 5.46, p < 0.001), while overall bias in visual localization toward proprioceptive targets decreased significantly (hand invisible, 24.0 ± 3.9%; hand visible, 4.4 ± 0.5%, paired t-test (19) = 22.29, p < 0.001). Finally, the discrepancy between visual and proprioceptive targets affected the localization bias as revealed by a significant three-way interaction between magnitude of spatial disparity, target modality, and hand visibility (F(3, 57) = 9.83, p < 0.001, η_p_^2^ = 0.34). Narrowing in on the bias found during reaches to visual targets illustrated in Fig. 3a,b (red curve), results demonstrate a significant interaction of hand visibility × magnitude of multisensory disparity (F(3, 57) = 14.35, p < 0.001, η_p_^2^ = 0.43). When the hand was not rendered (Fig. 3b), the magnitude of the disparity between proprioceptive and visual targets had a profound impact on reaching biases (F(3, 57) = 23.77, p < 0.001, η_p_^2^ = 0.56), while the magnitude of this disparity was inconsequential when the hand was rendered (F(3, 57) = 2.04, p = 0.11, 1 − β = 0.54, Fig. 3a). In the case of reaches toward proprioceptive targets (Fig. 3a,b, black curve), findings equally demonstrated a significant hand visibility × magnitude of multisensory disparity interaction (F(3, 57) = 3.849, p = 0.014, η_p_^2^ = 0.17). In this case, however, both when the hand was rendered (F(3, 57) = 43.59, p < 0.001, η_p_^2^ = 0.70) or not (F(3, 57) = 16.09, p < 0.001, η_p_^2^ = 0.46), the magnitude of visuo-proprioceptive disparity played a significant role. The effect of magnitude of spatial disparity on proprioceptive localization bias was, nonetheless greater in the case when the reaching hand was rendered than when it was not, as demonstrated by the fact that paired t-tests at each of the spatial magnitudes and across hand visibility conditions revealed significant differences at 25 mm (t(19) = 2.54, p = 0.020, Bonferroni-corrected) and 50 mm (t(19) = 2.46, p = 0.023, Bonferroni-corrected), but not at 100 mm (t(19) = 1.57, p = 0.132, Bonferroni-corrected) or 200 mm (t(19) = 1.61, p = 0.120, Bonferroni-corrected) visuo-proprioceptive disparity.

Regarding the endpoint dispersion in multisensory reaches, as shown in Fig. 3c,d results suggested that reaches toward the visual target was more precise than toward the proprioceptive target (S.D. = 7.6 ± 0.4 mm vs. 20.6 ± 2.2 mm; main effect of modality in a 2 (Hand visibility: Hand vs. No Hand) × 2 (Target Modality: Visual vs. Proprioceptive) × 4 (Visuo-proprioceptive disparity magnitude: 200 mm, 100 mm, 50 mm, and 25 cm) × 2 (Disparity direction: Leftward disparity vs. Rightward disparity) repeated-measures ANOVA; F(1, 19) = 38.24, p < 0.001, η_p_^2^ = 0.67). In addition, reaches were more precise when the hand was rendered than when it was not rendered (12.5 ± 1.1 mm vs. 15.7 ± 1.3 mm; main effect of hand visibility: F(1, 19) = 36.94, p < 0.001, η_p_^2^ = 0.66) but viewing the hand had different influences on the two target types (hand visibility × target modality; F(1, 19) = 80.67, p < 0.001, η_p_^2^ = 0.81), as viewing the hand improved precision in reaches toward visual targets (11.0 ± 0.7 mm vs. 4.2 ± 0.3 mm; paired t-test t(19) = 43.1, p < 0.001) but not toward proprioceptive targets (20.4 ± 2.3 mm vs. 20.7 ± 2.1 mm; paired t-test t(19) = 0.47, p = 0.64; Fig. 3d). The visuo-proprioceptive disparity magnitude × target modality interaction demonstrated a trend (F(3, 57) = 2.51, p = 0.067) while remaining non-significant, and the rest of interaction terms were not significant (all p > 0.322). We did not find consistent changes in precision as a function of visuo-proprioceptive discrepancy (F(3, 57) = 1.41, p = 0.249, 1 − β = 0.354) and disparity direction (F(1, 19) = 0.04, p = 0.835, 1 − β = 0.055).

### Model Fit

#### Coupling Prior Model

**Figure 4:**
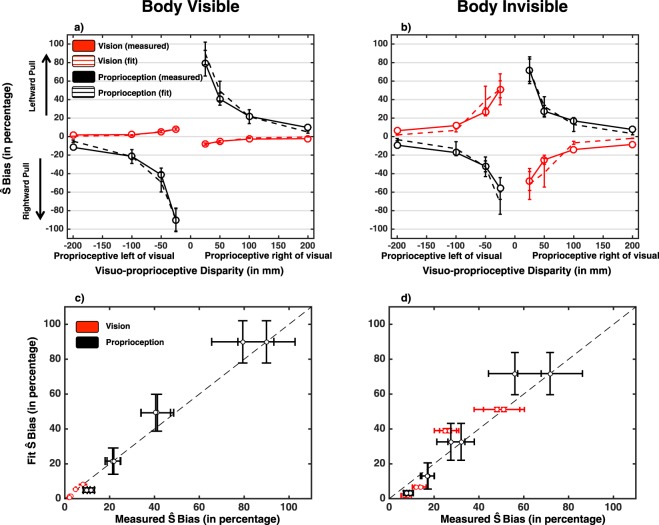
Coupling Prior Model of Visuo-Proprioception Location Estimates and Fit with Psychophysical Data. Top panels; average psychophysically measured (solid line) and modeled (dashed line) visuo-proprioceptive MAP-decoded location estimates after fitting λ and σ^2^_c_ and as a function of the placement of visuo-proprioceptive stimuli and hand visibility (left: visible; right: invisible). Proprioceptive stimuli (illustrated long the x-axis) are placed either 200 mm rightward, 100 mm rightward, 50 mm rightward, 25 mm rightward, 25 mm leftward (−25mm), 50 mm leftward (−50mm), 100 mm leftward (−100mm), or 200 mm leftward (−200mm) relative to the visual stimuli. Bottom panels: Fitted visual (red) and proprioceptive (black) bias (y-axis) as a function of measured bias (x-axis), for both when the hand was visually rendered (left) and not (right). Overall, there appears to be no systematic fitting error. Error bars represent +/−1 S.E.M.

Importantly, in addition to analyzing the accuracy and precision of reaching end-point error, data were analyzed within the coupling prior framework. The likelihood function associated with a visuo-proprioceptive reach was centered at the presented location with the dispersion fixed for each participant at their respective unisensory standard deviation for the corresponding condition (i.e., either hand visually rendered or not; see *Analyses: Coupling Prior* Section). The maximum of the posterior was equated to the participant’s response on each trial (i.e., MAP decoding), and thus we estimated the spatial dispersion of the visuo-proprioceptive coupling prior (i.e., σ_p_; coupling prior dispersion) as well as the relative weighting attributed to the spatially-specific coupling vs. the uniform distribution (i.e., λ; coupling prior strength) that best accounted for the data separately in the case when the reaching hand was rendered and not. As illustrated in Fig. 4, the model produced localization estimates that were closely in line with the psychophysical data. More specifically, the final average fits of the proprioceptive localization errors yielded an R^2^ = 0.77 (S.E.M. = 0.06) when the body was present, and R^2^ = 0.74 (S.E.M. = 0.05) when the body was not present. Importantly, these two conditions did not differ regarding goodness-of-fit (paired-samples t-test, t(19) = 0.50, p = 0.62). More in detail, residuals resulting from the coupling prior fitting were determined for each participant and experimental condition and analyzed via a 2 (Hand visibility: Hand vs. No Hand) × 4 (Visuo-proprioceptive disparity magnitude: 200 mm, 100 mm, 50 mm, and 25 cm) × 2 (Disparity direction: Leftward disparity vs. Rightward disparity) repeated-measures ANOVA. As illustrated in Fig. 4 (bottom panels), results show no systematic trend as evidenced by lack of significant main effect (Distance, F(3, 57) = 1.37, p = 0.26, 1 − β = 0.34; Direction, F(1, 19) = 0.034, p = 0.85, 1 − β = 0.054; Hand visibility, F(1, 19) = 0.042, p = 0.84, 1 − β = 0.054) and lack of any significant interaction (Distance × Direction, F(3, 57) = 0.52, p = 0.66, 1 − β = 0.151; Distance × Hand Visibility, F(3, 57) = 0.20, p = 0.89, 1 − β = 0.085; Direction × Hand Visibility; F(1, 19) = 1.88, p = 0.18, 1 − β = 0.25; Distance × Direction × Hand Visibility, F(3, 57) = 1.73, p = 0.17, 1 − β = 0.43).

#### Coupling Prior Parameters

**Figure 5:**
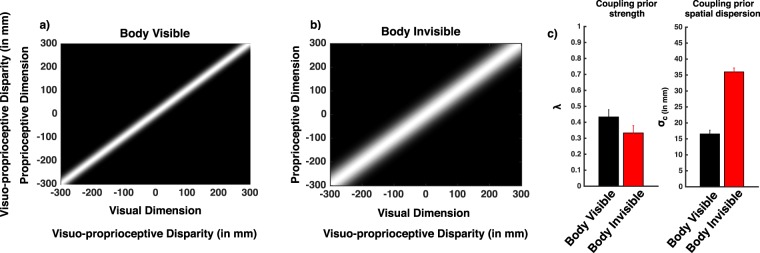
Parameters of the Best-fitting Visuo-proprioceptive Coupling Prior Model. The spread of the Gaussian that couples visual and proprioceptive information for the target hand (**a**) is wider if the reaching hand is visible compared to (**b**) when the hand is invisible. (**c**) Average λ (coupling strength) and ${\sigma }_{c}$ (coupling prior spatial dispersion indexed by the standard deviation) as a function of hand visibility. The general tendency to bind sensory information is decreased when the body disappears (left subpanel). Similarly, the spatial reliability of visuo-proprioceptive coupling is decreased when the hand is absent (vs. present; right subpanel). Error bars represent +/−1 S.E.M.

Lastly regarding the coupling prior framework, and most importantly, we analyzed the parameters resulting from the model fit, which appeared to successfully account for behavioral observations. This analysis (Fig. 5a,b) suggests that the spatial dispersion of the visuo-proprioceptive coupling was significantly affected by the presence or absence of a visual depiction of the hand (t(19) = 4.0, p < 0.001, Fig. 5c, right panel). The standard deviation of the Gaussian distribution describing the spatial profile of coupling between modalities increased when the hand was invisible (σ_c,invisible_ = 35.9 ± 5.0 mm) in contrast to when it was visible (σ_c,visible_ = 16.5 ± 1.2 mm). In contrast, participants’ weighting of spatially-specific vs. unspecific expectations remained unchanged (paired-sample t-test on λ t(19) = 1.32, p = 0.20, see Fig. 5c, left panel) when the hand was either present (λ_visible_ = 0.44 ± 0.04) or absent (λ_invisible_ = 0.34 ± 0.04). That is, the general exchange between integration and segregation was not statistically different when a visual hand was rendered during reaching or not.

#### Maximum-Likelihood Estimation Model

**Figure 6:**
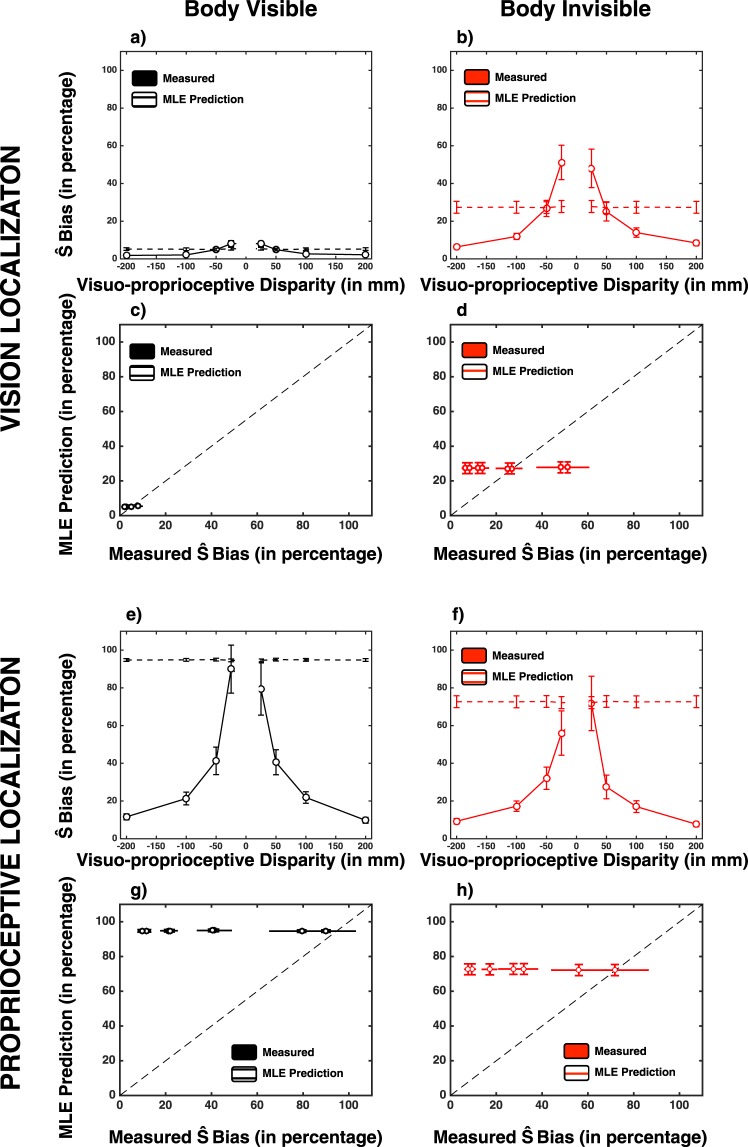
Predictions by Maximum-Likelihood Estimates Model and Fit with Psychophysical Data. (**a**,**b**) Psychophysically measured (solid line) and predicted (dashed line) visuo-proprioceptive bias (y-axis in percentage) in visual reaches as a function of the visuo-proprioceptive disparity and hand visibility. Proprioceptive stimuli (illustrated long the x-axis) are placed either rightward (positive values) or leftward (negative values) relative to the visual stimuli. (**c**,**d**) MLE-predicted visual bias (y-axis) as a function of measured bias (x-axis) expressed as a percentage of visuo-proprioceptive disparity. (**e**,**f**) Measured (solid line) and predicted (dashed line) end-point biases in reaches toward proprioceptive targets. (**g**,**h**) Predicted proprioceptive bias as a function of measured bias. Error bars represent +/−1 S.E.M.

In addition to casting PPS as a prior coupling visual and proprioceptive information, we contrasted visuo-proprioceptive location estimation to the predictions of a maximum-likelihood model (MLE) where the weighting of the components is proportional to their reliability^67^. This was undertaken both as a contrast to the coupling prior model, and in order to estimate whether visuo-proprioceptive coupling is “Bayes optimally integrated” under certain conditions. To perform this comparison using the same analysis as above, we forced fusion by setting λ = 1, and thus render Eq. 2 identical to Eq. 3, and we made the spatial dispersion around the diagonal close to zero by setting σ^2^_c_ = 10^−4^mm. In other words, we eliminate the spatial *gradient* to visuo-proprioceptive coupling. As illustrated in Fig. 6, the average fit of the MLE was poor (visual estimate hand visible: R^2^ = 0.41 ± 0.28; visual estimate hand invisible: R^2^ = 0.59 ± 0.31; proprioceptive estimate hand visible: R^2^ = 0.55 ± 0.33; proprioceptive estimate hand invisible: R^2^ = 0.59 ± 0.31), yet not different between visibility conditions (visual: paired-samples t-test: t(19) = 1.87, p = 0.07; proprioceptive: paired-samples t-test: t(19) = 0.70, p = 0.49).

More interestingly, residuals analysis revealed that the MLE model does not capture the entirety of responses for the various visuo-proprioceptive disparities, neither in visual and nor in proprioceptive reaches. Regarding the visual reaches, a 2 (Hand visibility: Hand vs. No Hand) × 4 (Visuo-proprioceptive disparity magnitude: 200 mm, 100 mm, 50 mm, and 25 cm) × 2 (Disparity direction: Leftward disparity vs. Rightward disparity) repeated-measures ANOVA on the MLE residuals (Fig. 6, top 4 panels) revealed no significant main effect (all p > 0.16), nor an interaction between variables (all p > 0.14). On the other hand, a similar 2 (Hand visibility: Hand vs. No Hand) × 4 (Visuo-proprioceptive disparity magnitude: 200 mm, 100 mm, 50 mm, and 25 cm) × 2 (Disparity direction: Leftward disparity vs. Rightward disparity) repeated-measures ANOVA on the MLE residuals for the proprioceptive reaches (Fig. 6, bottom 4 panels) revealed a significant main effect of visuo-proprioceptive magnitude disparity (F(3, 57) = 40.95, p < 0.001, η_p_^2^ = 0.68). All other main effects and interactions were non-significant (all p > 0.17). The main effect of disparity magnitude was driven by significant residuals present at disparities of 200 mm (one-sample t test to zero, p < 0.001), 100 mm (p < 0.001), and 50 mm (p < 0.001). In contrast, the MLE model predicted well proprioceptive bias when visual and proprioceptive targets were presented at a disparity of 25 mm (p = 0.437). This observation is in line with the fact that MLE is a forced-fusion model, and hence when participants are indeed binding visual and proprioceptive information, the MLE accounts well for observed localization, but it performs poorly under conditions were segregation is required (e.g., at large visuo-proprioceptive discrepancies). Interestingly, the fact that the MLE predicts well proprioceptive bias both when the reaching hand is visible and invisible when visual information is within 25 mm of the proprioceptive target suggests that behavior under these specific conditions reflects Bayes optimal integration. The contrast between MLE – where fits are good solely at near distances – and coupling prior fits – where fits are good over the entire spectrum of visuo-proprioceptive disparities suggests that the inclusion of a spatial gradient for coupling (i.e., the coupling prior) allows for appropriately accounting for multisensory integration throughout the near and far space.

## Discussion

Participants performed reaches toward a visual or a proprioceptive target in a virtual environment while their reaching hand was either visually rendered or not. In some conditions the targets were unisensory, while in other conditions both proprioceptive and visual targets were presented with a spatial disparity and participants were asked to reach toward each of them in succession. The variability and bias of their reaches were analyzed and compared to the predictions of a coupling prior and maximum likelihood model of multisensory integration in order to estimate (i) the relative weighting participants attributed to the spatially-specific vs. non-specific visuo-proprioceptive coupling, and (ii) the spatial dispersion of the spatially specific visuo-proprioceptive coupling prior, as well to establish (iii) whether visuo-proprioceptive integration was Bayes optimal at certain spatial disparities, and (iv) whether the inclusion of a coupling gradient allowed for appropriately accounting for visuo-proprioceptive integration throughout near and far space. Novelty, we propose that the parameter governing the spatial dispersion of the coupling prior (σ_c_) is related to the participants’ PPS.

Results suggest that reaches toward visual targets are more precise than reaches toward proprioceptive targets (by a factor of 2 to 4). Precision of reaches toward visual targets is decreased when the rendered reaching hand is removed whereas precision in reaching toward proprioceptive targets is unaltered by this manipulation. Consistent with this pattern of variability, when the body is visible and hence there is a greater difference between visual and proprioceptive reliabilities, reaches toward proprioceptive targets are more biased toward concurrently presented visual stimuli (see Figs 3 and 4, and^58,77^, for a similar observation across the audio and visual modalities). Lastly, under the coupling prior framework^65–69^, localization of visual and proprioceptive targets in this task is not solely influenced by the noisiness of participants sensory representations, but equally by their space-dependent expectation of the congruency between the location of visual and proprioceptive stimuli in the world; participants’ visuo-proprioceptive coupling prior. Interestingly, results suggested that the spread of the Gaussian function dictating the spatial specificity of visuo-proprioceptive coupling more than doubles when the reaching hand disappears. This suggests that there is a relaxation of the space-specific expectation for multisensory congruency when the body is invisible. Remarkably, the general strength attributed to the coupling prior remains unaltered by the presence or absence of the visual hand.

Although our results are qualitatively in line with seminal observations by van Beers and colleagues^59–61^ who demonstrated that relative reliabilities between vision and proprioception signals accounted for the final location estimate of visuo-proprioceptive targets, the similarity does not hold when several conflicts are employed as we did here. van Beers and colleagues employed a Maximum Likelihood Estimation framework^67^ that is meant to capture only conditions of complete fusion, and not the transition between multisensory integration and segregation as discrepancy between the signals increases. In other words, their model does not utilizes a parameter dictating the relative weighting between integration and segregation (here, λ), nor a parameter privileging certain spatial relations between visual and proprioceptive sensory information; latent variables that exist in our model and allow for better fit between model and behavioral observations by virtue of a slight increase in model complexity. Indeed, according to the MLE visuo-proprioceptive integration was seemingly “optimal” at small spatial discrepancies (up to 25 mm), but this model vastly overestimated coupling at further visuo-proprioceptive distances (from 50 mm onward). On the other hand, including a parameter allowing for the handoff between integration and segregation, as well as a spatial parameter dictating visuo-proprioceptive expectancies as a function of distance allowed for appropriately describing visuo-proprioceptive integration throughout (azimuthal) space. Interestingly, the spatial parameter was Gaussian – as opposed to linear, for instance – suggesting a true boundary between areas of space where visuo-proprioceptive coupling is strong (near space) vs. areas of space where this coupling is weak (far space). Arguably, the present findings – visuo-proprioceptive pull that is stronger in near as opposed to far space and that is well accounted by a Gaussian function (i.e., two sigmoidals, for the leftward and rightward visual discrepancies vis-à-vis the finger) – are reminiscent of the findings by Guterstam and colleagues^54^ suggesting a space-dependent attractive pull between touch applied on the hand and in mid-air near the hand. The current work expands on Guterstam and colleagues’ “perceptual correlate of visuo-tactile PPS”^54^ by proposing a computational framework and suggesting that the attractive pull, i.e., the inference from touch in space to touch on the body, can be accounted by a coupling prior framework. Additionally, while Guterstam and colleagues’ study focused on the PPS surrounding the hand (and in a vertical direction), our study delineated the attractive pull specifically surrounding an index finger (and in a horizontal direction). These results add to the existing literature indexing PPS surrounding the hand^26,29^, legs^77^, face^19,21,78^, trunk^8,17,20,35^, and now fingers.

In addition to accounting well for visuo-proprioceptive integration as a function of spatial disparity, the inclusion λ and σ_c_ within the coupling prior framework, in conjunction with the data-fitting approach undertaken here, allows for the scrutiny of variables that are inaccessible to direct measurement. Indeed, a main interest here resided in the putative impact that the presence or absence of a visual depiction of a virtual hand may have on the underlying visuo-proprioceptive coupling prior. In fact, we suggest that PPS can be conceived to be a ‘stochastic bubble’ surrounding the body and computing the probability that an object will come in contact with the body (see^56^ for similar arguments). As such, in principle, the measurement of the strength of the link between body-related information (i.e., proprioceptive signals) and surrounding exteroceptive signals (i.e., visual) arguably provides an index for the computation of proximity to the body that may characterize the functionality of PPS. The observation that the spatial specificity of the coupling prior between visual and proprioceptive sensory modalities drastically reduced upon the disappearance of the body can hence be interpreted as the PPS becoming ill defined when the body disappears (see^19,79^ for a similar argument), a finding that makes a good deal of ecological sense. In turn, these results seemingly suggest that the visual presence of a body is an important constituent in constructing a PPS representation – and perhaps a bodily self-consciousness - yet importantly, it is not a necessary and sufficient one. Indeed, as mentioned above, previous reports have reported that the process of multisensory integration within the PPS may inclusively lead to the embodiment of empty volumes of space (the invisible hand illusion^43^; illusory ownership over an invisible body^42^). Here we suggest that illusions of ownership over empty spaces are likely possible due to the fact that although no body is present, the invisible body^42^ or body-part^43^ may retain a PPS representation (see^53^) that albeit weakened still leads to the scaffolding of a sense of body ownership^20,35^. Taken together, the observation that invisible bodies preserve a PPS representation, and that embodiment over invisible bodies is possible^42,43^ further argues for the putative role of PPS in bodily self-consciousness^33,34^.

To further confirm this conclusion in the future it will be important to replicate the findings of the current report while equally measuring PPS via other “traditional” approaches. That is, as argued by several different other authors^44,45,51,79^, it is possible that different PPS representations exist, and the interrelation between multisensory PPS defined via reaction times^25,26^, PPS as defined by defensive reflexes^57^, reaching space^41,80^, and now the method developed here, is unclear. It is unequivocal that here we demonstrate a multisensory coupling that is space-dependent, yet how this spatial extent relates to, say, the spatial region within which tactile reaction times are facilitated^27^ (a more classic definition of PPS) remains an open question. Similarly, recent studies have suggested that while PPS is body-part specific, these different PPS representations interact with one another. Thus, while the results here suggest that 200 mm is outside the peri-finger space – in that there is no visuo-proprioceptive coupling – it is also true that all targets here were presented within reaching limit (due to task constraints). Thus, all targets were presented within the reaching limit, and putatively within the peri-trunk space^25^. In the future it will be interesting to examine how being within vs. outside different PPS representations (e.g., hand, face, trunk) interacts with the space-dependent attractive pull toward the finger described here.

Lastly, the finding that the visual rendering of the hand does not only significantly impact reaching accuracy and precision, but equally alters the spatial expectation that multisensory stimuli co-occur in space is highly relevant in the understanding of virtual reality (VR), where the world and bodies do not merely exist, but have to be recreated. Results regarding the localization error and spatial dispersion of visual and proprioceptive targets highlight that as visual renderings in virtual reality become increasingly sophisticated, realistic, and reliable, an equal effort has to be put toward generating tactile and proprioceptive experiences to make the experience believable. The sole focus on rendering exteroceptive sensory modalities such as audition and vision risks to further and further “pull” proprioceptive estimates of the body in VR toward audio/visual objects that are more faithfully rendered. On the other hand, the finding that not visually depicting the human body relaxes the internal visuo-proprioceptive coupling of signals may be of significance in scenarios where visuo-proprioceptive incongruence can have a functional advantage^81,82^. Regardless, a fundamental component of our daily life is undoubtedly the presence of a physical body we own, and hence ultimately replicating this experience ought to be a major goal in VR. Seemingly, body ownership is at least partially reliant on the successful integration of sensory stimuli from distinct modalities, and hence preserving the conditions necessary for the process of multisensory integration, such as appropriate visuo-proprioceptive spatial expectancies, is an imperative.
